# Genomics of the relict species *Baronia brevicornis* sheds light on its demographic history and genome size evolution across swallowtail butterflies

**DOI:** 10.1093/g3journal/jkad239

**Published:** 2023-10-17

**Authors:** Alba Marino, Eliette L Reboud, Emmanuelle Chevalier, Marie-Ka Tilak, Jorge Contreras-Garduño, Benoit Nabholz, Fabien L Condamine

**Affiliations:** Institut des Sciences de l'Evolution de Montpellier (Université de Montpellier | CNRS | IRD | EPHE), Place Eugène Bataillon, 34095 Montpellier, France; Institut des Sciences de l'Evolution de Montpellier (Université de Montpellier | CNRS | IRD | EPHE), Place Eugène Bataillon, 34095 Montpellier, France; Institut des Sciences de l'Evolution de Montpellier (Université de Montpellier | CNRS | IRD | EPHE), Place Eugène Bataillon, 34095 Montpellier, France; Institut des Sciences de l'Evolution de Montpellier (Université de Montpellier | CNRS | IRD | EPHE), Place Eugène Bataillon, 34095 Montpellier, France; Universidad Nacional Autónoma de México, Escuela Nacional de Estudios Superiores, campus Morelia, Antigua Carretera a Pátzcuaro #8701, Col. Ex-Hacienda San José de la Huerta, 58190 Morelia, Michoacán, Mexico; Institut des Sciences de l'Evolution de Montpellier (Université de Montpellier | CNRS | IRD | EPHE), Place Eugène Bataillon, 34095 Montpellier, France; Institut Universitaire de France (IUF), Paris, France; Institut des Sciences de l'Evolution de Montpellier (Université de Montpellier | CNRS | IRD | EPHE), Place Eugène Bataillon, 34095 Montpellier, France

**Keywords:** conservation genomics, genome size, papilionidae, relict species, transposable elements

## Abstract

Relict species, like coelacanth, gingko, tuatara, are the remnants of formerly more ecologically and taxonomically diverse lineages. It raises the questions of why they are currently species-poor, have restrained ecology, and are often vulnerable to extinction. Estimating heterozygosity level and demographic history can guide our understanding of the evolutionary history and conservation status of relict species. However, few studies have focused on relict invertebrates compared to vertebrates. We sequenced the genome of *Baronia brevicornis* (Lepidoptera: Papilionidae), which is an endangered species, the sister species of all swallowtail butterflies, and is the oldest lineage of all extant butterflies. From a dried specimen, we were able to generate both long-read and short-read data and assembled a genome of 406 Mb for *Baronia*. We found a fairly high level of heterozygosity (0.58%) compared to other swallowtail butterflies, which contrasts with its endangered and relict status. Taking into account the high ratio of recombination over mutation, demographic analyses indicated a sharp decline of the effective population size initiated in the last million years. Moreover, the *Baronia* genome was used to study genome size variation in Papilionidae. Genome sizes are mostly explained by transposable elements activities, suggesting that large genomes appear to be a derived feature in swallowtail butterflies as transposable elements activity is recent and involves different transposable elements classes among species. This first *Baronia* genome provides a resource for assisting conservation in a flagship and relict insect species as well as for understanding swallowtail genome evolution.

## Teaser

Sequencing the genome of *Baronia brevicornis*, an endangered species and the oldest butterfly lineage, revealed a high level of heterozygosity but a sharp population decline in the past million years. As the sister species of all swallowtail butterflies, this study also highlights genome size variation in Papilionidae and the role of transposable element activity in their evolution. The *Baronia* genome provides valuable resources for understanding swallowtail genome evolution and assisting in conservation efforts.

## Introduction

Since Darwin (1859), relict species have captivated evolutionary biologists, who often view them as curious ‘living fossils’ or remnants of old times. Some plants such as *Ginkgo* (*ca.* 80 million years ago, Ma, Royer et al. 2003) and vertebrates such as the *Solenodon* (*ca.* 60 Ma, Brace et al. 2016), and the coelacanth (*ca.* 75 Ma, Cavin and Guinot 2014) are perhaps the most famous relict organisms. Because of their ancestry, they are thought to provide interesting and important information on a vanished past and can be used to understand the evolution of clades and biotas (Grandcolas et al. 2014). Relict species raise the questions of why they are currently depauperate, have restrained (highly specialized) ecology, and are vulnerable to extinction (often ranked as endangered).

**Fig. 1.**
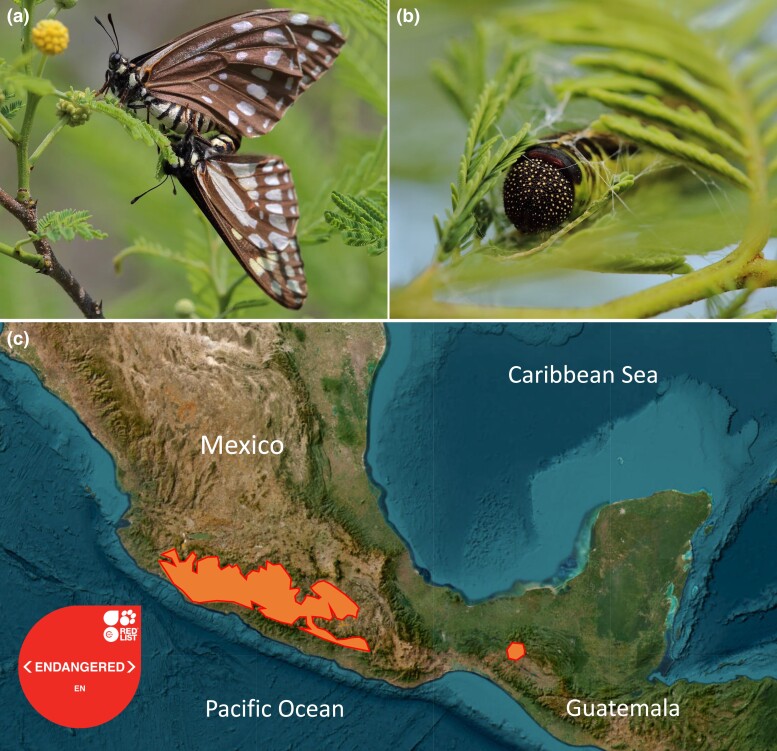
a) Two adults of *B. brevicornis* mating. b) A caterpillar of *B. brevicornis* hiding in a shelter built with leaves of its host plant *Vachellia cochliacanha* (formerly *Acacia cochliacanha*). c) The distribution range of *B. brevicornis* is composed of two populations, shown in orange. Photos: Jorge Contreras-Garduño. The map has been designed on MapMaker: https://mapmakerclassic.nationalgeographic.org based on data from the IUCN Red List https://www.iucnredlist.org/ (Puttick et al. 2018).

Among invertebrates, the swallowtail butterfly *Baronia brevicornis* (Salvin, 1893) (Lepidoptera: Papilionidae) is considered a relict species and is regarded as one of the most mysterious butterfly species in the world (Fig. 1a). Molecular phylogenetic and phylogenomic studies have shown that *B. brevicornis* is the sister species of all swallowtail butterflies (Condamine et al. 2012; Espeland et al. 2018; Allio, Scornavacca, et al. 2020). Depending on divergence time estimates from phylogenies, *B. brevicornis* diverged about 55–75 Ma (Condamine et al. 2012; Allio, Scornavacca, et al. 2020), and is the oldest lineage of all extant butterflies, which originated about 100 Ma (Espeland et al. 2018; Chazot et al. 2019; Kawahara et al. 2019). It has been argued that the lineage leading to *B. brevicornis* survived the Cretaceous/Paleogene mass extinction and has maintained a relatively unchanged morphology at least for about 80–90 Ma (Heikkilä et al. 2012; Machkour-M’Rabet et al. 2014; Legal et al. 2015; Galicia-Mendoza et al. 2021). One of the most interesting features of *B. brevicornis*, contrary to all other Papilionidae, is that their caterpillars feed strictly on *Vachellia cochliacantha* (formerly *Acacia cochliacantha*: Fabaceae; Fig. 1b). According to some results, Fabaceae would be the ancestral host-plant preference of all butterflies (Kawahara et al. 2023) and potentially swallowtail butterflies (Simonsen et al. 2011 but see Condamine et al. 2012; Allio et al. 2021), which highlights the importance of studying *Baronia* to understand early evolution of butterflies.

Although the geographic distribution of its host plant is large, *B. brevicornis* has a restricted natural range (León-Cortés et al. 2004). *B. brevicornis* is strictly endemic to Mexico within tropical dry forests (Machkour-M’Rabet et al. 2014; Legal et al. 2015; Galicia-Mendoza et al. 2021), located in central southern Mexico, including the Rio Balsas (Balsas River) drainage basin that extends from the western state of Jalisco to the southern state of Oaxaca (Fig. 1c). There is also a small population in the state of Chiapas, specifically in the Central Valley, close to the city of Tuxtla Gutiérrez (Fig. 1c). Based on this disjunct distribution (more than 450 km) between the central Mexico and Chiapas populations, previous authors have proposed the presence of two subspecies: the nominal *B. brevicornis* (Salvin, 1893) and *B. brevicornis rufodiscalis* (De la Maza and White, 1987) for the isolated populations of Chiapas. Due to its particular phenotype, mysterious origin, and localized distribution, *B. brevicornis* has been listed as a near threatened (NT) species since 1986 by the IUCN Red List of Threatened Species but has been recently ranked as endangered (EN) by the IUCN (Puttick et al. 2018, Fig. 1).

Despite being a remarkable and taxonomically important species, knowledge about the ecology and host-plant use has remained very scarce until recently (Machkour-M’Rabet et al. 2014; Legal et al. 2015; Galicia-Mendoza et al. 2021). Yet, natural history data on this species are needed to help establish conservation programs. Some studies have shown that *B. brevicornis* populations cannot occur if the host plant does not cover at least two-thirds of the locality, and that even in the most favorable zones, the occupancy of the butterfly does not exceed 2.5% of the available habitat even when its host plant covers 50% of the area (Legal et al. 2015). Moreover, it has been suggested that the locality where *B. brevicornis* resides should provide not only host plants for the caterpillars but also food sources for the adult butterflies (Galicia-Mendoza et al. 2021). A few molecular studies have assessed the genetic diversity of *B. brevicornis*. Machkour-M’Rabet et al. (2014) have found high genetic diversity within the species. These high values can be explained by the population dynamic of this species characterized by a very high density of individuals over very limited areas (Legal et al. 2015), while inter-population variation is thought to reflect both the age of colonization and locality perturbation level. Genetic structure analysis further revealed three populations, suggesting that both habitat and host-plant specificity probably limit the exchange of individuals between populations.

With the advent of genomic data and analyses, several studies have addressed a range of evolutionary questions using relict species as biological models. For example, the coelacanth genome has been instrumental to reveal tetrapod evolution (Amemiya et al. 2013) or to elucidate how ancestral aquatic vertebrates evolved into terrestrial animals (Nikaido et al. 2013). In addition, genomes of relict and endangered species can also be used to assist conservation actions. For instance, assessing genome-wide diversity provides a good proxy for the loss of adaptive potential that an endangered species has experienced. Similarly, inferring demographic history of an endangered species has emerged as a powerful tool for unveiling possible past bottlenecks in the population dynamics (Nadachowska-Brzyska et al. 2015). Estimation of genome-wide diversity and demography can be estimated by computing heterozygosity and inbreeding depression obtained from whole-genome analyses (Robinson et al. 2022). However, these analyses require generating a relatively high-quality whole genome to obtain less biased estimates of heterozygosity.

Within swallowtail butterflies, whole-genome sequencing has allowed addressing several questions in evolutionary biology and ecology such as identifying molecular adaptations during host-plant shifts (Allio et al. 2021), studying the origin and evolution of color diversity (He et al. 2022), or investigating population genomic and demographic history (Reboud et al. 2023). The availability of swallowtail genomes also brings the question of genome size evolution across the family (Liu et al. 2020) as we have gleaned knowledge from genome assembly (e.g. Allio, Scornavacca, et al. 2020; Podsiadlowski et al. 2021; He *et al*. 2022). It appears that the subfamily Parnassiinae shows large genome sizes (between 500 Mb and 1.4 Gb), while the subfamily Papilioninae has smaller genome sizes in general (around 300 Mb but can go up to 930 Mb in *Graphium*). Therefore, *B. brevicornis*, as the sister subfamily to all other swallowtails, stands out as a key lineage to study genome size evolution across the family Papilionidae. The draft genome of *B. brevicornis* published by Allio, Scornavacca, et al. (2020) is around 480 Mb but is only assembled with short-read data at low depth of coverage, which impedes an accurate estimate of its genome size and restrains demographic inferences as well as computation of heterozygosity level.

In this study, we present a first genome assembly of *B. brevicornis* generated with a long-read sequencing approach, complemented with genomic polishing using short-read and homology-based annotation. Using this newly assembled genome, we (1) assess the level of nuclear heterozygosity to be compared with other swallowtail butterflies, (2) estimate the demographic history of the species to correlate with past events (in particular climate fluctuations) by taking into account the ratio of recombination rate over mutation rate, and (3) provide insights into the genome evolution across swallowtail butterflies by annotating transposable elements (TEs) for 18 available high-quality whole genomes of Papilionidae. This reference genome will enable future population genomic studies with *B. brevicornis*.

## Materials and methods

### Sample, library preparations, and sequencing

In May 2019, we collected a single specimen of the nominal species *B. brevicornis*, an adult female (voucher FC897), coming from the Northern population of Mexico (Morelos state, south of Mexico City). The specimen was dried and stored in a freezer at −20°C without any additional preservation product. Tissues from the thorax were used to extract high-molecular weight DNA. Following Reboud et al. (2023) who tested two different extraction methods, we used the Qiagen genomic DNA kit to obtain a better 260/230 ratio as estimated with Nanodrop assays guaranteeing DNA purity for long-read sequencing with Oxford Nanopore Technology (ONT). Final DNA purity and concentrations were measured using both Nanodrop (Thermo Fisher, USA) and Qubit (Thermo Fisher, USA).

Whole-genome libraries were constructed using the resulting high-molecular-weight DNA as input for the Nanopore LSK-109 ligation kit (Oxford Nanopore Technologies, UK) following the manufacturer's protocol. Long-read sequencing was performed on a GridION device with three R9.4.1 flow cells. Remaining DNA extractions were sent to Novogene Europe (Cambridge, UK) for two library preparations. Libraries were generated using NEBNext DNA Library Prep Kit following manufacturer's recommendations and indices were added to each library. Genomic DNA was randomly fragmented to a size of 350 bp by shearing, then DNA fragments were end-polished, A-tailed, and ligated with the NEBNext adapter for Illumina sequencing, and further PCR enriched by P5 and indexed P7 oligos. The PCR products were purified (AMPure XP system) and the resulting libraries were analyzed for size distribution by Agilent 2100 BioAnalyzer and quantified using real-time PCR. Since the genome sizes for *Baronia* was estimated to be about 480 Mb (Allio, Scornavacca, et al. 2020), Illumina 150 bp paired-end sequencing was run on a NovaSeq 6000 instrument to obtain about 30 Gb per library corresponding to a genome depth-of-coverage of >100× after combining the two libraries.

### Genome assembly

For ONT sequencing, all raw long-read sequence data (fast5 files) were basecalled using Guppy 5.0.15 with the super-high accuracy mode and a quality control of 10 (min_score 10). Sequencing adapters were trimmed using Porechop 0.2.3 (https://github.com/rrwick/Porechop). Draft genome assemblies were performed with the long-read assembler Flye 2.8.3 (Kolmogorov et al. 2019, https://github.com/fenderglass/Flye) with default options. The Illumina raw reads were cleaned, filtered, and paired using fastp 20.0 (Chen et al. 2018) with default options. To improve base accuracy and reduce assembly errors, the long-read Flye draft assemblies were polished with POLCA (Zimin and Salzberg 2020) implemented in MaSuRCA 4.0.1 (Zimin et al. 2013). Assembly statistics were then assessed using the gVolante2 platform (Nishimura et al. 2017) to retrieve the number and size of contigs, the presence, completeness, and duplication of BUSCO genes of the Lepidoptera *odb10* database (Manni et al. 2021). We also checked for haplotype duplication using the spectra-asm plot with Merqury (Rhie et al. 2020). Merqury identifies the number of times each k-mer identified in the reads is present in the assembly allowing an assessment of whether some k-mers are present twice or more in the assembly, indicating the presence of haplotype duplication. Before submitting genomes assemblies to GenBank, we checked for possible contaminations using BlobTools 1.1.1 (Laetsch and Blaxter 2017) set to the ncbi and diamond databases. We found no evidence of artificial contamination coming from laboratory manipulation, but some contigs were clearly identified as belonging to exogenous organisms such as host plants and symbionts (Supplementary Figure 1). We removed all contigs that were belonging to Bacillota (formerly Firmicutes) (all belonging to Order Lacterobacillales) or Pseudomonadota (all belonging to Order Enterobactererales) phylum (Supplementary Table 1).

We used GetOrganelle 1.7.7 (Jin et al. 2020), with the cleaned and paired short-reads and a *B. brevicornis* reference mitogenome from a previous study (GenBank accession number: LT999970, Condamine et al. 2018) to assemble a new *B. brevicornis* mitogenome. The resulting assembly was given to MitoFinder 1.4 (Allio, Schomaker-Bastos, et al. 2020) to annotate and extract protein-coding genes, tRNA and rRNA genes. The new mitogenome of *B. brevicornis* is OR063968. This mitogenome was aligned with the mitogenome of *B. brevicornis* LT999970 (Condamine *et al*. 2018) and variable sites were counted with the “Statistics” option in Seaview (Gouy et al. 2010).

### Genome annotations and whole-genome alignment

We performed the MAKER2 pipeline of gene annotations. First, the repeat sequences were reconstructed using RepeatModeler 2.0.1 (Flynn et al. 2020). These newly identified repeats were used to annotate the repeat sequences using RepeatMasker (Smit et al. 2015) in association with the Dfam libraries (Storer et al. 2021) setting the parameter “–species Arthropoda”. Second, we ran MAKER 2.31.11 (Holt and Yandell 2011) using the repeat annotated by RepeatMasker and homology information obtained with the protein sequences of *Heliconius melpomene*, *Melitaea cinxia*, *Papilio machaon*, *Papilio xuthus*, and *Papilio glaucus*. Third, SNAP (Korf 2004) and AUGUSTUS (Stanke et al. 2006) were used to produce gene prediction models from the first round of MAKER. BUSCO 5.5 (Simão et al. 2015) with options “–long” and “–augustus” and the Endopterygota database was used to produce the gene prediction model of AUGUSTUS. Finally, we ran again MAKER using the annotation from the first round and the gene models of SNAP and AUGUSTUS to produce a final round of annotations.

We also used the BRAKER2 pipeline, specifically designed to perform annotation for assembly without RNA-seq data (Brůna et al. 2021). This pipeline uses the assembly soft-masked for the repeat sequences and a large set of coding sequences from distantly related species to perform the annotation. Here, we used the Arthropoda set of OrthoDB 11 (Kuznetsov et al. 2023) available at https://bioinf.uni-greifswald.de/bioinf/partitioned_odb11/ in which we added the sequences of *P. xuthus* and *Ornithoptera alexandrae*.

As quality control, we evaluated the mismatches between the hemizygous Z contigs and Illumina read consensus. We performed a genome assembly with the Illumina reads only using MEGAHIT (Li et al. 2015). We then aligned the MEGAHIT contigs onto our long-read-based assembly using Minimap2 (Li 2018). Alignments were filtered based on maximal quality (60) and must represent more than 80% of the MEGAHIT contig size. We then estimated the divergence between the Z contigs and the Illumina reads.

Finally, we performed whole-genome alignments using Progressive Cactus (https://github.com/ComparativeGenomicsToolkit/cactus) (Armstrong et al. 2020) with the genomes of *P. bianor* (Lu et al. 2019), *P. machaon* (Lohse et al. 2022), and *O. alexandrae* (Reboud et al. 2023). The contigs with less than 10% of their size aligned to Z or W chromosomes were excluded. This alignment will allow identifying sequences of *Baronia* genome assembly that are homologs to chromosome Z and W of *Papilio* assemblies and Z of *Ornithoptera* assemblies.

### Nuclear heterozygosity

Genotype calling was performed for both Illumina and Nanopore data. For Illumina data, read mapping was performed using the SpeedSeq pipeline (Chiang et al. 2015) that relies on BWA (Li 2013) excluding duplicated reads using SAMtools (Li et al. 2009). Genotype calling was performed using Freebayes 1.3.2 (Garrison and Marth 2012). We excluded positions with a coverage below 15× and higher 200×. SNP with a quality below 200 were excluded. For ONT data, read mapping was performed using Minimap2 (Li 2018) and genotype calling with LongShot 0.4.1 (Edge and Bansal 2019), using a threshold of 10× minimum and 150× maximum for the depth of coverage (minimal quality of 50) and applying a transition/transversion rate for genotype prior estimation (*ts_tv_ratio*) of 2.0 (Edge and Bansal 2019). SNP with a quality below 300 as provided by Freebayes in the VCF were excluded. For ONT, a SNP is considered only if the alternate allele is supported by at more than 20% of the reads and less than 80% of the reads. We used python programs VCF2fasta_no_mono.py and VCF2FastaLongshot.py (https://github.com/benoitnabholz/VCF2Fasta) to convert the VCF file into fasta files for Illumina and ONT data, respectively. Next, sites annotated as repeat by RepeatModeler/RepeatMasker were excluded. Finally, heterozygosity was computed on the whole genome using the program heterozygosity.py (https://github.com/benoitnabholz/popgen_python) or focusing on BUSCO single-copy orthologs to compute synonymous and 4-fold degenerated heterozygosity using the programs *seq_stat_coding* (https://github.com/benoitnabholz/seq_stat) and *selectClassSite* (https://github.com/benoitnabholz/selectClassSite).

### Estimation of the demographic history and effective population size

We relied on a sequential Markovian coalescent (SMC) model (Schiffels and Wang 2020) to estimate the ancestral effective population size (*Ne*) of *Baronia*. The SMC model needs to be calibrated, in particular with a value of mutation rate. However, it has been shown that SMC models do not perform well when the ratio of recombination rate (*r*) over mutation rate (*μ*) becomes greater than one (Sellinger et al. 2021). This phenomenon seems to be prevalent in invertebrate genomes. Assuming a single crossover per tetrad per male meiosis and 30 chromosomes, Reboud et al. (2023) have estimated that the average recombination rate for *O. alexandrae* (a swallowtail butterfly) is 2.7e−8, which is more than 10 times higher than its average mutation rate estimated at 1.316e−9 mutations per site per generation. To investigate the sensitivity of SMC analyses to parameters, Reboud et al. (2023) have simulated data with the range of recombination and mutation rate parameters with *r* being 10 times higher than *μ* such as those observed in invertebrates. Fitting a SMC model with default options (*-rhoOverMu = 0.25*) vs an adjusted ratio of *r* over *μ* (-*rhoOverMu = 10*) recovered different demographic histories, with the model using the adjusted ratio showing a good fit to the simulated data (Reboud et al. 2023). Accordingly, the ratio of *r* over *μ* must be well adjusted to recover trustworthy demographic inferences in invertebrates.

It is likely that *Baronia* has a mutation rate close to *Ornithoptera* as it is also in the range of the mutation rates estimated for *Heliconius* (Nymphalidae) between 1.3e−9 and 5.5e−9 (Keightley et al. 2015). In addition, *Baronia* is the only species in the subfamily Baroniinae, which is sister to all remaining Papilionidae (Allio, Scornavacca, et al. 2020, Allio 2021), and is thus too distantly related to any extant species to be able to accurately estimate the mutation rate using neutral divergence. Therefore, we used the same mutation rate of *O. alexandrae* (*μ* = 1.3e−9). Assuming *B. brevicornis* genome is 406 Mb long, distributed in 30 chromosomes and that there is a single crossover per tetrad per male meiosis, the recombination rate would be *r* = 3.38e−8.

We relied on the multiple sequentially Markovian coalescent (MSMC) model as implemented in MSMC2 (Schiffels and Wang 2020; https://github.com/stschiff/msmc2). We used the VCF files generated using Longshot (as described in the *Nuclear heterozygosity* section) and created the so-called “mask file” for each individual based on the depth of coverage thresholds of >20× and <150× using a custom python script (available in FigShare). These files were then combined using the “generate_multihetsep.py” of MSMC2 to generate “multihetsep.txt” input files (https://github.com/stschiff/msmc-tools/blob/master/msmc-tutorial/guide.md). We followed the recommendations of Reboud et al. (2023) using MSMC2, which was thus set with the option *−rhoOverMu = 10* to better account for the ratio *r* over *μ* that is higher than 10. Contigs shorter than 500 kb and of the Z chromosomes were excluded representing a total of 171 Mb (42% of the genome size). We generated 10 bootstraps using the multihetsep_bootstrap.py (https://github.com/stschiff/msmc-tools) and generated all graphs with the R package ggplot2 (Wickham 2016) by considering a generation time of 1 for *B. brevicornis* (Legal et al. 2015).

### Transposable elements and repeat sequence dynamics

TEs were annotated for the genome assemblies of *Baronia* and 17 other high-quality genomes of swallowtail butterfly species (Li et al. 2015; Nishikawa et al. 2015; Liu et al. 2019; Podsiadlowski et al. 2021; He *et al*. 2022; Mackintosh et al. 2022; Reboud et al. 2023; see Supplementary Table 2 for details). We used Earl Grey 1.2 (https://github.com/TobyBaril/EarlGrey), which is a pipeline combining several tools for TE detection and performing automated consensus curation. Firstly, repeats in the assembly are masked with RepeatMasker 4.1.2 by homology to known metazoan sequences from Dfam 3.5 and RepBaseRepeatMaskerEdition-20181026 (-r metazoa). RepeatModeler 2.0.3 is then employed on the hard-masked genome to search novel TE families. The new detected sequences undergo an iterative process of consensus refinement and elongation (-i 5 -f 1000, default options): this aims to reduce the redundancy and improve the quality of the *de novo* library. Finally, the unmasked genome is mined with LTR_finder 1.0.7 and again with RepeatMasker using the combined curated *de novo* and public metazoan libraries. The resulting annotations are merged and defragmented and overlappings removed. For a detailed description of the pipeline see Baril et al. (2022). Earl Grey computes the genetic distance between the annotated TE copy and their respective consensus sequences. Consensus sequences correspond to a reconstruction of the ancestral sequences of the TEs and, therefore, the genetic distance could be used as an estimation of the age of the TE insertion in the genome. To compare the genetic distance between the TEs and their consensus sequences and the divergence among species, we compute the genetic distance among the species using the same method. The alignments of TEs to their consensus produced by the second RepeatMasker run were extracted with a custom script (available in FigShare) and used to compute Kimura 2P distance (i.e. Kimura K80) copy by copy with the *dist.dna* function of the R package ape 5.7-1 (Paradis and Schliep 2019). To estimate the divergence among species, we used the orthologous sequences provided by BUSCO 5.5 (Manni et al. 2021) selecting the 524 genes present in all species. BUSCO genes were aligned using the OMM_MACSE pipeline that is a codon aware alignment method (Ranwez et al. 2018; Scornavacca et al. 2019). Third-codon positions were extracted from the alignment and the same pairwise genetic Kimura 2P distance between species was computed. A phylogeny of the 18 species was obtained from Allio, Scornavacca, et al. (2020) and Allio (2021) and associated with the obtained median genetic distances. The interspecies distances were then compared to the genetic distances between TEs and their consensus to determine if TE insertions were anterior or posterior of species divergences.

## Results and discussion

### Genome sequence statistics

For one adult female specimen collected in 2019, stored in dry conditions, we sequenced the DNA combining a mean of 54× of long reads (Oxford Nanopore, 32 Gb: N50 = 3,027 bp, the mean = 2,160 bp, the median = 1,585 bp, and 1.16 Gb of reads have a length >10 kb) for draft assembly and 141× of short reads (Illumina, 56.9 Gb) for polishing (see *Materials and Methods*). Using Flye assembler (Kolmogorov et al. 2019) and POLCA polisher (Zimin and Salzberg 2020), we assembled the genome of *B. brevicornis* that is 406 Mb, which has 4,834 contigs (after removing 152 contigs that were detected as exogenous) and a N50 of 0.4 Mb (Table 1). Over a total of 5,286 core genes of the Lepidoptera database (*odb10*, Manni et al. 2021), BUSCO recovered 93.7% single complete genes, 1.1% duplicated genes, 1.3% fragmented genes, and 3.9% missing genes (Table 1). The very low proportion k-mers present in the Illumina reads were present twice or more in the assembly confirming a very low proportion of haplotype duplication in the assembly (Supplementary Figure 2). The genome size and gene completeness of our *B. brevicornis* assembly has a lower quality though fairly comparable to previously published genomes of swallowtail species: *Papilio demoleus* (Papilionini: 240 Mb, BUSCO recovered 98.1% single complete genes, 0.2% duplicated genes, 0.7% fragmented genes, and 1.0% missing genes), *Troides helena* (Troidini: 330 Mb, BUSCO recovered 95.9% single complete genes, 0.3% duplicated genes, 0.5% fragmented genes and 3.3% missing genes), *Lamproptera curius* (Leptocircini: 550 Mb, BUSCO recovered 89.3% single complete genes, 0.2% duplicated genes, 1.7% fragmented genes, and 8.8% missing genes), or *Parnassius orleans* (Parnassiini: 1.18 Gb, BUSCO recovered 92.2% single complete genes, 1.1% duplicated genes, 1.2% fragmented genes, and 5.5% missing genes), which were assembled with similar data and methods (He *et al*. 2022). Furthermore, the genome size of *Baronia* stands between the generally small genomes in the subfamily Papilioninae and the large genomes mostly found in the subfamily Parnassiinae (Liu et al. 2020; He *et al*. 2022), which brings questions on the evolutionary dynamic of genome size across the family Papilionidae. Gene annotation with MAKER and BRAKER2 leads to 14,362 and 17,747 annotated protein-coding sequences of mean length 7,150 bp and 6,703 bp, respectively (available in FigShare). The BUSCO scores for the protein sets were 93.7% complete including 91.1% single and 2.6% duplicated for BRAKER2 and 92.5% complete including 90.0% single and 2.5% duplicated for MAKER.

**Table 1.** Assembly statistics for the genomes of *B. brevicornis*.

	Raw data sequenced (Gb) (LR + SR)	Final mean coverage (LR + SR)	Assembly size (bp)	Number of contigs	N50 (bp)	Max length (bp)	Number of gaps (≥5 N's)	BUSCO score (%)
**Voucher 167 (** Allio, Scornavacca, et al. 2020 **)**	0 + ∼11	0 + ∼23x	488,028,434	973,148	1,169	33,471	35,404	S:50.5; D:0.5; F:19.2; M:29.8
**Voucher FC897 (this study)**	31.96 + 56.9	54x + 141x	405,627,949	4,834	412,514	2,621,275	39	S:93.7; D:1.1; F:1.3; M:3.9

Amount of raw data and final mean coverage of Voucher 167 were approximated from Allio, Scornavacca, et al. (2020). LR, long reads; SR, short reads; bp, base pairs. For BUSCO scores, S, single-copy genes; D, duplicated genes; F, fragmented genes; M, missing genes out of 5,286 genes in *odb10* lepidopteran database.

### Identification of sex chromosomes

We sequenced a female, allowing us to identify the Z/W chromosomes based on coverage and heterozygosity information. Median coverage after read cleaning and excluding mapping position quality lower than 50.0 and base quality lower than 30.0 leads to 54× for ONT data and 75× for Illumina data (after removing PCR duplicates). The classification was made difficult due to the strong correlation between GC content and depth of coverage observed with both ONT and Illumina data (Supplementary Figure 2). However, a visual inspection of the relationship allows identifying two categories of contigs with one category having a lower depth of coverage considering their GC content (contigs above the line in Supplementary Figure 2). These contigs are probably from the sex chromosome. This is confirmed by the lower heterozygosity (=3e−4) and 76.3% of the contigs (148 out of the 194) have no SNP. Finally, contigs that have been aligned to the Z chromosome of *P. bianor* (contig no. 30) and of *P. machaon* all fall in this category. In contrast, none of the five contigs (392 kb in total) aligned to the W chromosome of *P. machaon* show a pattern of coverage or genetic diversity compatible with a haploid status, thus corresponding to autosomal regions (Supplementary Figure 3). One *Baronia* specimen was previously sequenced, but at low (25×) coverage, and was an adult male (NCBI accession SRR8954515; Allio, Scornavacca, et al. 2020). We compared this male with the present female specimen to identify and exclude a few more contigs that belong to the Z chromosome (Supplementary Figure 4; in total 193 contigs representing 18 Mb).

Finally, the haploid Z chromosome sequence provides an opportunity to evaluate our genome quality by quantifying the mismatch between the Illumina reads and the Z contigs. We found a median divergence of 0.02%, corresponding to two differences per 10 kb, between the contigs obtained using the Illumina reads and the Z contigs of long-reads assembly. Assuming that this divergence provides an estimate of the assembly error rate, it is 25 times lower than the estimated heterozygosity level.

### *Baronia* has unexpectedly elevated genomic diversity levels

Using Illumina data and annotation with MitoFinder, the *Baronia* mitogenome was reconstructed with a base coverage of 468× and we retrieved all genes including the 13 protein-coding genes and 2 rRNA genes. Comparing this new mitogenome with the previous mitogenome of the same population (LT999970), the mitogenomic diversity (π-diversity) including coding and noncoding regions was calculated at ∼1.6% (with 236 variable sites over 14,728 aligned positions). For comparison, the mitochondrial diversity of the endangered butterfly *O. alexandrae* is 22 times lower (∼0.0704%; Reboud et al. 2023) than that of *B. brevicornis*. Recovering an elevated mitogenomic diversity for an endangered species is unexpected, in particular when comparing individuals of the same population. However, it is difficult to conclude with mitogenomic evidence only because we lack a large-scale estimation of mitochondrial diversity based on whole mitogenomes for butterflies since studies estimating mitochondrial diversity usually relied on the cytochrome *c* oxidase subunit I DNA barcode marker (e.g. Dincă et al. 2021 for European butterflies). In addition, high mitochondrial diversity does not necessarily equate to high autosomal diversity (Mackintosh et al. 2019).

Excluding the Z chromosome and contigs shorter than 30 kb, Illumina and ONT data lead to very similar estimates of autosomal heterozygosity around 0.59% and 0.56%, respectively. Heterozygosity is highly variable among contigs. Even selecting contigs larger than 200 kb, heterozygosity varies from less than 0.25% to 1.0% among contigs. Focusing on the BUSCO single-copy orthologs, synonymous nucleotide diversity is 0.78%, nucleotide diversity of 4-fold degenerated position is 0.75% (*n* = 4,533 genes), and the ratio of nonsynonymous nucleotide diversity over synonymous nucleotide diversity (pN/pS) is 0.136. Compared to other butterflies, the genetic diversity of *Baronia* is relatively low (Mackintosh et al. 2019) but is much higher than other endangered swallowtail butterflies (Reboud et al. 2023).

Our understanding of the determinants of heterozygosity remains debated (e.g. Ellegren and Galtier 2016; Mackintosh et al. 2019). Neutral genetic diversity is proportional to effective population size and mutation rate. Therefore, range size, endangered status or life-history traits may influence *Ne* and be associated with the level of genetic diversity (e.g. Romiguier et al. 2014; Mackintosh et al. 2019; Buffalo 2021). Given the specific features of *B. brevicornis* like its relict status, ancestral habits, and endangered status, one may be tempted to explain the inferred heterozygosity level. Indeed, the endangered swallowtail *Luehdorfia taibai* has a restricted distribution range in the Qinling Mountains in China (Fang et al. 2019) and shows a very low heterozygosity level of 0.057%, similar to that of the giant panda (Guan et al. 2022). However, it seems that species’ specific traits do not correlate well with heterozygosity levels in swallowtail butterflies (Mackintosh et al. 2019). For instance, in the threatened troidine swallowtails (*O. alexandrae*, *O. priamus*, and *Troides oblongomaculatus*), there are heterogeneous levels of heterozygosity ranging from a very low level (0.0737% autosomal, 0.0704% neutral for *T. oblongomaculatus*) to a medium level (autosomal 0.433%, neutral diversity 0.708% for *O. priamus*). Within the species-rich genus *Papilio*, these heterozygosity levels are much higher with estimates ranging from 1.0% to 2.3% even if estimated with a different method (Lu et al. 2019). Therefore, our estimate of the heterozygosity level for *Baronia* cannot be explained by the species’ range or body size that could have an indirect effect on *Ne* or mutation rate, as suggested in Mackintosh et al. (2019).

In a genetic study, Machkour-M’Rabet et al. (2014) also found a high level of heterozygosity and suggested that the peculiar population dynamic of *Baronia* in high population densities over very restricted areas can explain such a high genetic diversity at the species level. This important density of individuals would favor significant intra-population genetic diversity over a long period of time. Finally, *Baronia* is well known for its color polymorphism with three male and three female color wing morphs observed in the Northern population (Galicia-Mendoza et al. 2021). This raises the question of: How are multiple morphs maintained in local populations against genetic drift that leads to loss of genetic variation and recombination that breaks up nonrandom trait associations? Balancing selection such as negative frequency-dependent selection (rare-morph advantage) or over dominant selection (heterozygote advantage) could be at play. One possible research avenue would involve a population genomic study to link this color polymorphism with fundamental evolutionary processes like frequency-dependent selection, gene flow, recombination, and to better understand the maintenance of heterozygosity level. With more genomic data, it would also be possible to compare with other butterfly cases, such as in the genus Papilio (Kunte et al. 2014; Timmermans et al. 2017), to better understand the origin and maintenance of sex-limited morphs.

### Sharp decline in the demographic history of *Baronia* in the last million years

We estimated how the genetic diversity translates into the demography dynamic for the species. Given the extinction risks and the relict status of *Baronia*, we expected a prolonged decline of effective population size such as those inferred for the endangered Queen Alexandra's birdwing butterfly (*O. alexandrae*, Reboud et al. 2023) or the Apollo butterfly (*Parnassius apollo*, Kebaïli et al. 2022); the latter has population declines throughout different mountain massifs even with high heterozygosity levels. However, recent studies have also unveiled long-term low but stable effective population size for endangered species such as in the vaquita porpoise (Morin et al. 2021; Robinson et al. 2022). Except the cases on *O. alexandrae* (Reboud et al. 2023) and *P. apollo* (Kebaïli et al. 2022), there are still few examples of demographic history in insects, other than pest insects (but see Walton et al. 2021; Manthey et al. 2022; García-Berro et al. 2023).

**Fig. 2.**
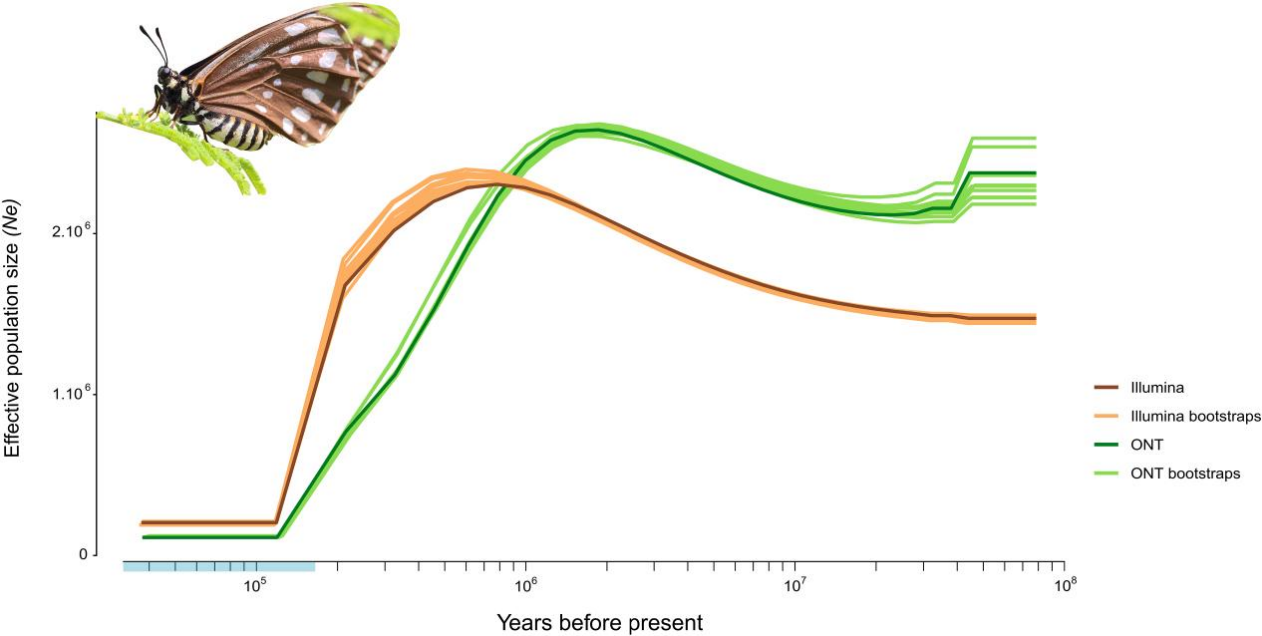
Estimated historical demography of *B. brevicornis*. MSMC2 estimates of the effective population size (*Ne*) with both Illumina and Nanopore data. Bootstraps are represented in clear lines. MSMC2 analyses were performed with *-rhoOverMu = 10* to consider a recombination rate 10 times higher than the mutation rate. The discrepancy between the Illumina and ONT datasets is likely due to the repeated sequences (Supplementary Figure 5). The pale blue rectangle along the time bar indicates the limits of the last glaciation period in the late Pleistocene. Photo: Jorge Contreras-Garduño.

Demographic analyses with MSMC2 traced relatively similar demographic histories for *Baronia* with Illumina and ONT data analyzed independently (Fig. 2; Supplementary Figure 5). The effective population size *Ne* of *Baronia* seems to have been at a low but continuously increasing number from ∼1,500,000 to 2,500,000 in effective population size from its origin until the last million years, when the *Ne* reached its maximum. However, we inferred a drastic demographic decline in the last million years or so (from ∼900,000 years with Illumina data to ∼2 million years with ONT data) that stabilized to low *Ne* (less than 250,000 individuals) around 120,000 years ago until the present (Fig. 2). This demography dynamic resembles that of endangered vertebrate species such as the brown hyena (Westbury et al. 2018) or the Californian condor (Robinson et al. 2021), whose effective population sizes were more elevated but declined in the last million years. Altogether, these results suggest that the ancestral effective population of *Baronia* has been larger than today, thus suggesting that its distribution range was probably larger.

Temporal variations of effective population sizes are usually compared with past climatic fluctuations such as temperature and/or sea level, in line with Quaternary glaciations (e.g. Nadachowska-Brzyska *et al*. 2015; Westbury *et al*. 2018; Morin et al. 2021). We can tentatively associate the inferred *Ne* variations of *Baronia* with the Pleistocene glaciation cycles, although it remains difficult to extract a correlation because of climatic heterogeneity and uncertainties on demographic parameters estimates. The last million years was mostly a glacial period that has also been documented in Mexico (e.g. Vazquez-Selem and Heine 2004). A drop in temperature of 5–9°C supports marked cooling over tropical land and oceans during the Pleistocene (Vazquez-Selem and Heine 2004). The cooling period coincides with the decrease of *Ne* for *B. brevicornis*.

Effective population sizes have also been compared with conservation status from IUCN data (Nadachowska-Brzyska *et al*. 2015; Wilder et al. 2023). It has been suggested that historical demography can inform contemporary resilience. For instance, mammal species with small historical effective population sizes show a large burden of deleterious alleles due to long-term accumulation and fixation of genetic load and have a higher risk of extinction (Wilder et al. 2023). The strong demographic contraction inferred with both types of data for *Baronia* is in line with the ideas that historical population size can be relevant to contemporary extinction risk and that genomic information can help predict extinction risk.

### Genome size evolution in Papilionidae

**Fig. 3.**
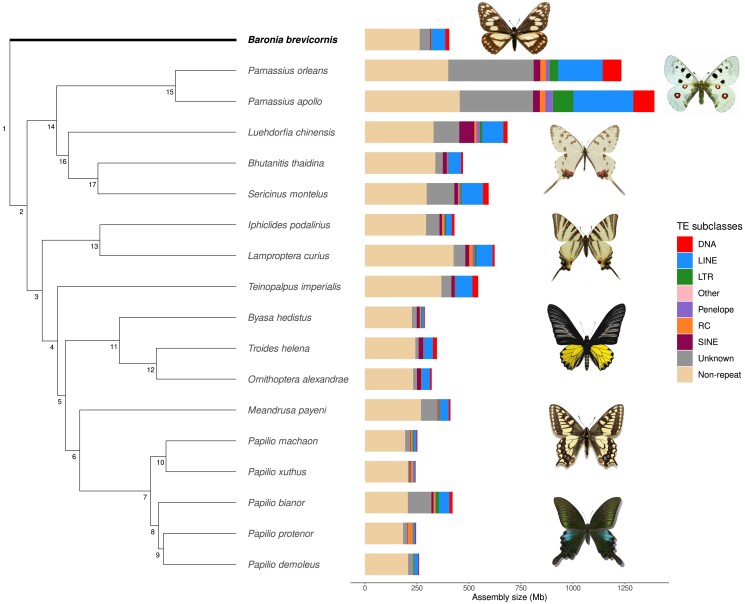
Variation in genome sizes (Mb) and transposable elements content across papilioninae, parnassiinae, and the monotypic Baroniinae represented by *B. brevicornis*. Node numbers correspond to the divergence points between species calculated as median pairwise Kimura 2P distance and reported in Fig. 4 and Supplementary Figures 3–6. The phylogeny was extracted from Allio, Scornavacca, et al. (2020) and Allio (2021). “Other” includes simple repeats, microsatellites, and RNA sequences. Photos: Fabien L. Condamine.

Genome size is extensively variable among species of Papilionidae (Liu et al. 2020; Podsiadlowski et al. 2021). The largest assembly (*P. apollo*, 1,392 Mb) is 5.7 times larger than the smallest genome (*P. xuthus*, 244 Mb). In accordance with other genomic studies (Petersen et al. 2019; Wu and Lu 2019; Cicconardi et al. 2023), this variation is largely explained by TEs (Pearson's correlation coefficient = 0.98; correlation coefficient under phylogenetic generalized least squares = 0.47, *P* < 0.0001), with the genomes of *P. apollo* and *P. xuthus* composed of 67% and 15% of TEs, respectively. However, the nonrepeat fraction of the genome is also variable among species and is not closely following overall genome size. For example, *L. curius* has a genome of 623 Mb with only 32% of annotated TEs whereas the similar size *Sericinus montela* (594 Mb) has 50% of TEs (Fig. 3;Supplementary Table 2). This can be due to ancient TEs persisting in the genome: indeed, our analysis is limited to relatively young TEs that could be recognized as such, while it is not able to recover old TEs which have substantially diverged from their ancestral sequence. However, the fact that genome size variation is mostly explained by TE content suggests that unannotated TE relics probably just moderately contribute to such variation. Another limit lies in the quality of the genome: as repeated sequences are the main responsible for gaps, the fragmented assembly of Baronia could hinder the recovery of its complete TE complement. However, the genome size estimated using GenomeScope is close to our assembly size (380 Mb for GenomeScope and 405 Mb for the assembly), so it is perhaps unlikely that a large proportion of TE sequences is missing.

Among the known TE subclasses, we find long interspersed nuclear elements (LINEs) to be the most abundant across all species (11% of the genome assembly on average), followed by DNA and short interspersed nuclear elements (SINEs) (3%). At the same time, a high variability of TE landscapes is generally reported across and within insect orders (Petersen et al. 2019; Gilbert et al. 2021): this is also found at the level of swallowtail butterflies as some species show lineage-specific element expansions as compared to their closer relatives. For instance, *Papilio protenor* displays an increase in rolling circle (RC) elements (9%), *Luehdorfia chinensis* has 11% of its genome covered by SINEs, and *P. apollo* genome underwent an expansion of long terminal repeat elements (LTRs) (7%). It should be noted that in many cases unknown elements take up a relevant portion of the genome, partly limiting our understanding of TE variability. Indeed, unknown elements are putative TE sequences that were identified as repeated but could not be classified by the pipeline, and correspond in broad terms to new, lineage-specific TE families. Because manual curation of *de novo* TE libraries was not performed, in some cases unknown sequences might be however spurious hits resulting, for example from gene duplications (Goubert et al. 2022).

Given that genome size has a patchy distribution across the phylogeny of Papilionidae and TEs appear to be major determinants of genome size variation, the condition of the common ancestor of swallowtail butterflies is uncertain: current genome size differences in this group could have arisen either through a differential erosion of a genome originally replete with TEs, or through derived TE expansions inflating genomes in certain lineages.

**Fig. 4.**
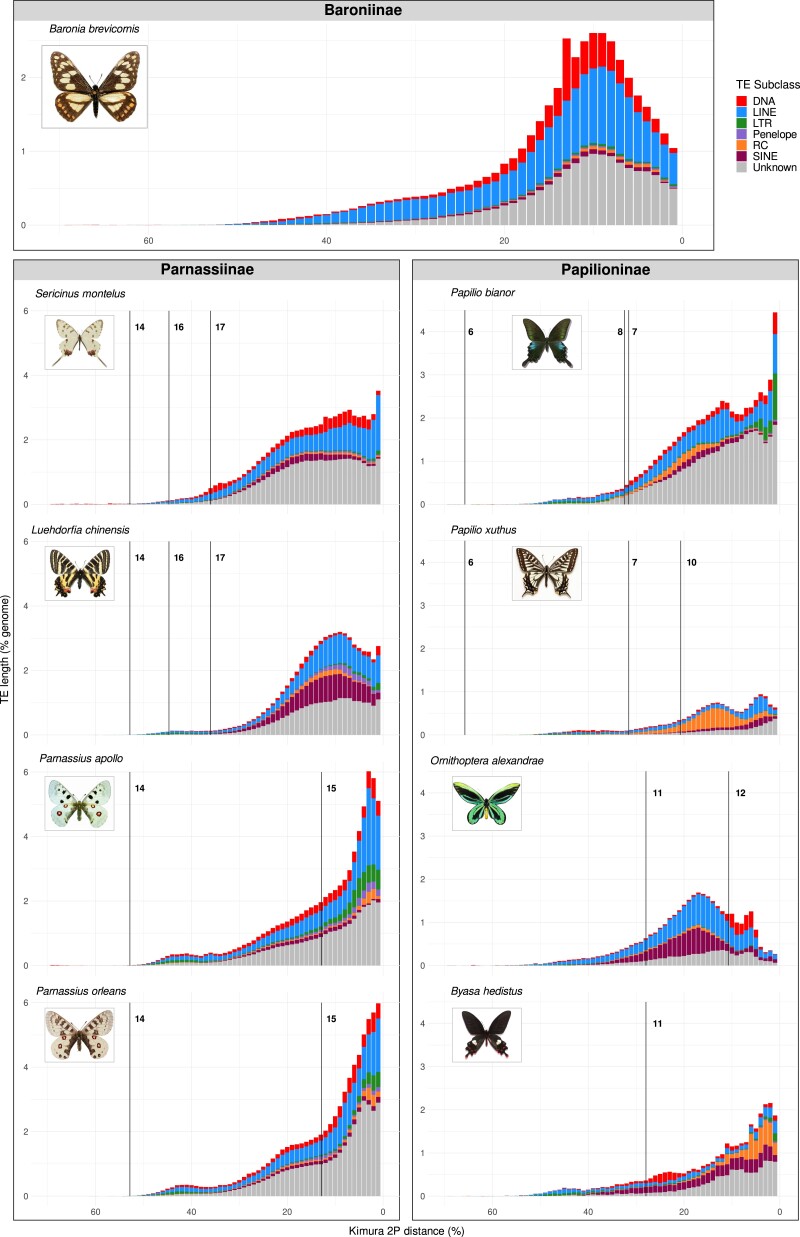
Distribution of TE copies in genome percentage according to their divergence from consensus. *Baronia*'s landscape is shown along with some of the main lineages from the two other subfamilies whose landscapes can be compared by the divergence nodes shown in Fig. 3. Low distance copies (right side of the plots) correspond to recently active ones, while highly divergent copies (left side of the plots) are remnants of old TE activity. Photos: Fabien L. Condamine.

To understand whether the contribution of TEs to genome size variation is more recent or ancient, we investigated the distribution of TE copies’ ages according to their distance from the “parental” sequence that originated upon transposition in each species. This analysis was coupled with the assessment of the approximate divergence between species to pinpoint shared and lineage-specific patterns of TE expansion. Because elements diverging by more than 50% from the consensus generally cannot be detected, TE landscapes could be compared only between the most closely related species (Fig. 4; Supplementary Figures 6–9). Despite their modest sizes, most of the TEs in the genomes of Papilionini seem to have originated independently after lineage splits in all species: RC mostly characterizes the recent activity in *P. xuthus* and *P. protenor* genomes, while LINEs are mainly active in the other species (Fig. 4; Supplementary Figure 6). Within Troidini, mainly LINEs and SINEs were active before the split of the three species (node 11, 28% divergence), after which *B. hedistus* lineage accumulated TEs independently from *O. alexandrae* and *T. helena* which share a clear TE burst before their separation (node 12, 11% divergence) (Fig. 4; Supplementary Figure 7). As for Zerynthiini and Luehdorfiini, most of the TEs seem to have accumulated independently in each lineage (node 17, 36% divergence) (Fig. 4; Supplementary Figure 8). Finally, in the two *Parnassius* genomes both common and novel TE expansions can be observed. Overall, the TE profiles of all species point at a mostly recent and lineage-specific TE activity as responsible for the present genome size variation across swallowtail butterflies and strongly suggest that big genomes such as those of Parnassiini are derived. That being said, it should be noted that this analysis provides landscapes of relatively recent TEs, which are able to explain current genome size variation in swallowtail butterflies. Indeed, we found that the TE activity is recent or ongoing even in Troidini, in the genera *Papilio* and in *Baronia*, which all have typically small-sized genomes. As it is clear that TE dynamics actively fuel current genome plasticity, we cannot rule out similar TE outbreaks that we are not able to track down with current methods to have shaped the early genome evolution of Papilionidae. Moreover, it should be emphasized that changes in genome size are the result of both repeat sequences expansion and deletion processes acting jointly (Kapusta et al. 2017): the high turnover rate of elements reported in Nymphalidae (Lavoie et al. 2013; Baril and Hayward 2022), and the reconstruction of both genomic expansion and shrinkage events along the evolution of the same family (Cicconardi et al. 2023) support this view.

Why the genomes of some groups tend to be refractory to repeat accumulation, while others do not, remains an open question. Lynch and Conery (2003) proposed that TEs might be able to accumulate in lineages with low effective population size as an effect of the reduced efficacy of selection against these mildly deleterious mutations. The reduced polymorphism associated with increased genome size in some populations of the genus *Leptidea* agrees with this view (Talla et al. 2017). However, no relationship between genetic diversity and genome size was observed when considering a wide set of lepidopteran species (Mackintosh et al. 2019). Small-RNA-based epigenetic regulation is known to be an important and widespread mechanism put in place by the host to tame selfish element proliferation (Blumenstiel 2011). As horizontal transfer of TEs seems to occur very frequently in Lepidoptera (Reiss et al. 2019), this might be an important factor repeatedly facilitating genome size variation in this group. Indeed, it has been suggested that foreign elements coming from distantly related taxa could be able to elude the host defense machinery and more promptly invade the new genome (Schaack et al. 2010 ; Venner et al. 2017). Given the dynamicity of their genomes, swallowtail butterflies are therefore an ideal group to investigate the evolutionary mechanisms underlying genome size variation.

### Limitations

Because of the preservation in dry conditions of the specimen, we could not sequence a greater amount of long reads data and transcriptomic data that would have helped to assemble a more contiguous genome with a proper RNAseq-based annotation. As a result, our genome has a fairly low N50 and is more fragmented, compared to previous genome assemblies in Papilionidae based on similar types of data (e.g. He *et al*. 2022; Reboud et al. 2023). However, this genome still represents an interesting resource for this rare and endangered species that fills a knowledge gap in this insect family. Although we recovered a similar demographic trend for *Baronia* between Illumina and ONT data, our MSMC inferences show some level of discrepancy, which should then be interpreted with caution and better understood. By replicating the MSMC2 analyses with the repeat masked, we found that the Illumina and ONT datasets produce similar demographic trends (Supplementary Figure 5), suggesting that the discrepancy between the Illumina and ONT datasets is due to the repeat sequences. The ONT are likely to be mapped more accurately on the repeat portion of the genome and excluding these regions leads to a homogenization of all results. Despite lineage-specific features unveiled, we also remain careful on our results and interpretations of genome size evolution because a more accurate genus-level sampling within Parnassiinae and Papilioninae can bring new information.

## Conclusion

In this study, using a combination of long and short reads, we presented the genome of *Baronia brevicornis*, an endangered butterfly species. The genome was found to be of comparable quality to other published genomes of swallowtail species. The heterozygosity level was found to be unexpectedly elevated, given its endangered status. However, the genetic diversity of this butterfly was found to be relatively low compared to other butterflies, but much higher than other endangered swallowtail butterflies. The historical demography is characterized by a strong decline of the effective population size initiated in the last million years that stabilized to a low effective population size in the last 100,000 years. As the sister species of swallowtail butterflies and the oldest lineage of all butterflies, the *Baronia* genome was pivotal to study genome size variation in Papilionidae. The activity of TEs is the primary driver of genome size evolution in swallowtail butterflies, indicating that the emergence of large genomes is a recent characteristic due to various TE classes. Overall, this study provides important insights into the genome and genetic diversity of this endangered butterfly species.

## Supplementary Material

jkad239_Supplementary_Data

## Data Availability

The genome, mitogenome, and sequencing data of the present study, including Nanopore, Illumina, and DNA data are available from the Genome database and Sequence Read Archive under the Bioproject accession number PRJNA971161, with the corresponding BioSamples accession number SAMN35020972, genome accession number JASFAT000000000. The mitogenome of *Baronia brevicornis* is OR063968. The genome annotations made with the BRAKER2 and MAKER pipelines, the masked reference genome, the scripts, and datasets to compute 4-fold degenerate site nucleotide diversity are available in the FigShare online repository: https://doi.org/10.6084/m9.figshare.22793513.v2. Supplemental material available at G3 online.
