# Supplementary material for: Genomics of the relict species Baronia brevicornis sheds light on its demographic history and genome size evolution across swallowtail butterflies
Source: G3 (Bethesda). 2023 Oct 17;13(12):jkad239. doi: 10.1093/g3journal/jkad239 (PMC10700114; doi:10.1093/g3journal/jkad239)
Supplement: jkad239_Supplementary_Data [file jkad239_supplementary_data.zip › Supplemental_Material_G3-2023-404604.pdf]

Supplemental Material

Figure S1. BlobTools plot for the genome assembly of *Baronia brevicornis*.

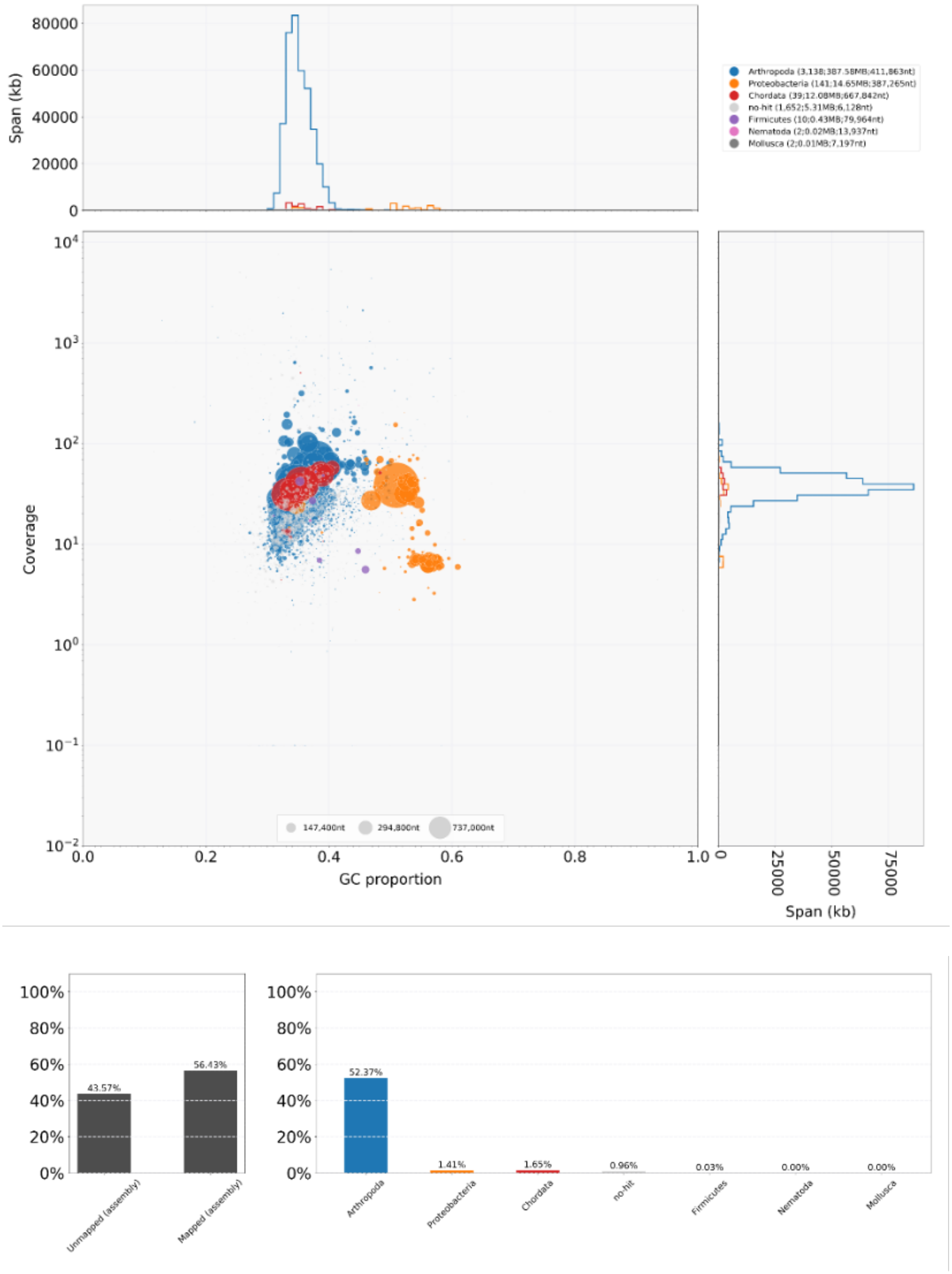

Figure S2. Spectra asm plot in Merqury.

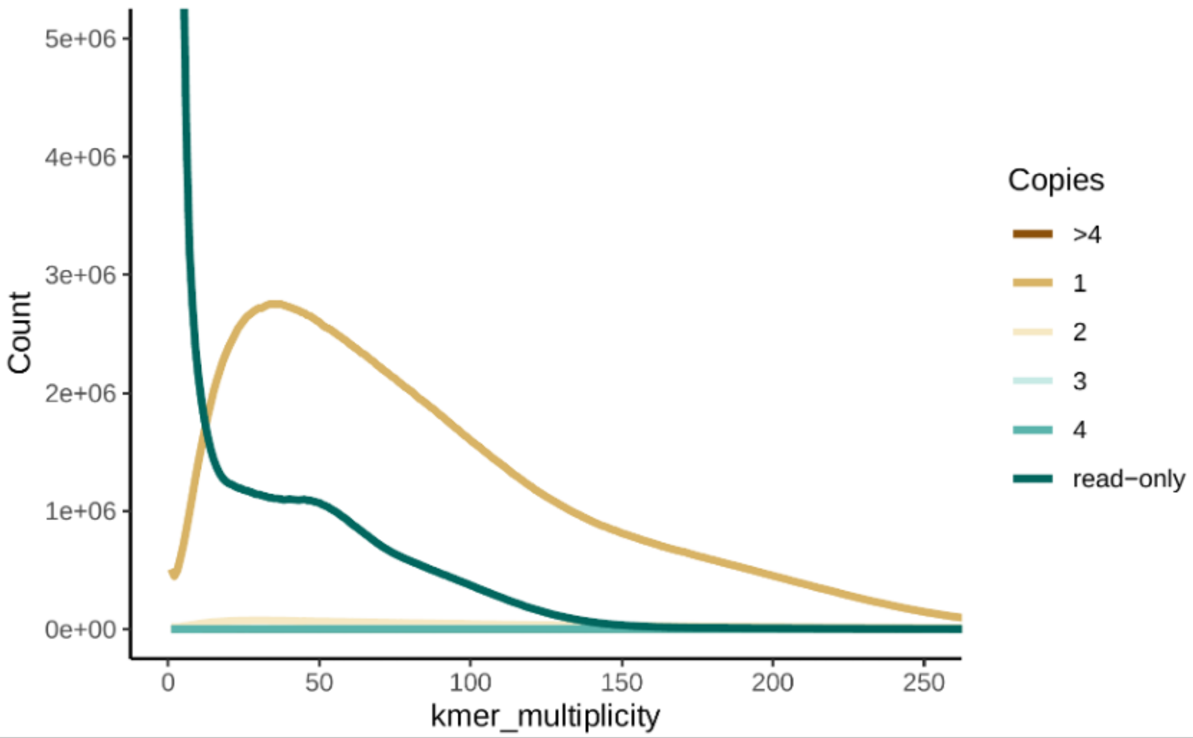

Figure S3. Plots of depth of coverage as a function of GC content per contigs. Only contigs longer than 30 kb are represented. A) Illumina data. The dotted line separates the two categories of contigs. Contigs above the line and with a coverage < 82x are probably from the Z chromosome. The purple points are the contigs that have been aligned to the Z chromosome (contig 30) of *Papilio bianor* and *P. machaon* using Cactus. The yellow points are the contigs that have been aligned to the W chromosome of *P. machaon*. B) Oxford Nanopore Technology data. The green points are the contigs that have been identified as the Z chromosome using the Illumina data.

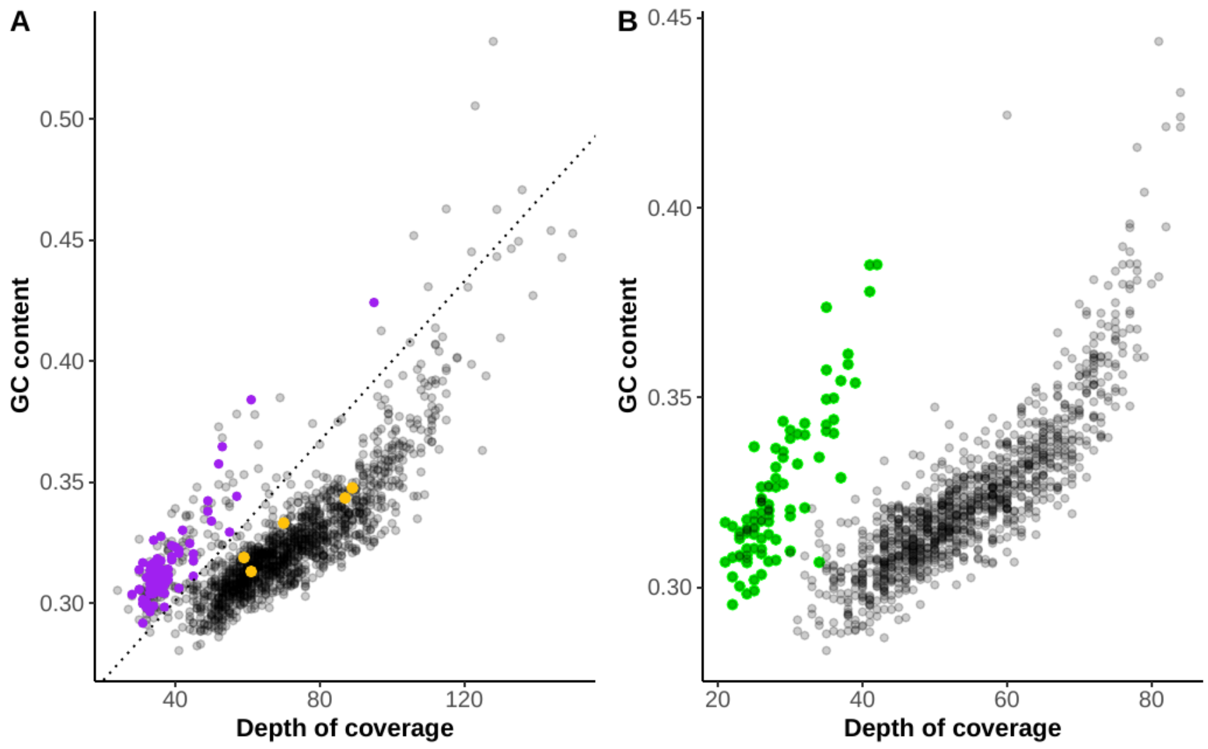

Figure S4. Heterozygosity of the contigs larger than 30 kb computed for the current genome assembly of a female and compared to the male sequenced in Allio *et al.* (2020a). Yellow points indicate the contigs belonging to the Z chromosome, while gray points indicate the contigs belonging to the autosomes.

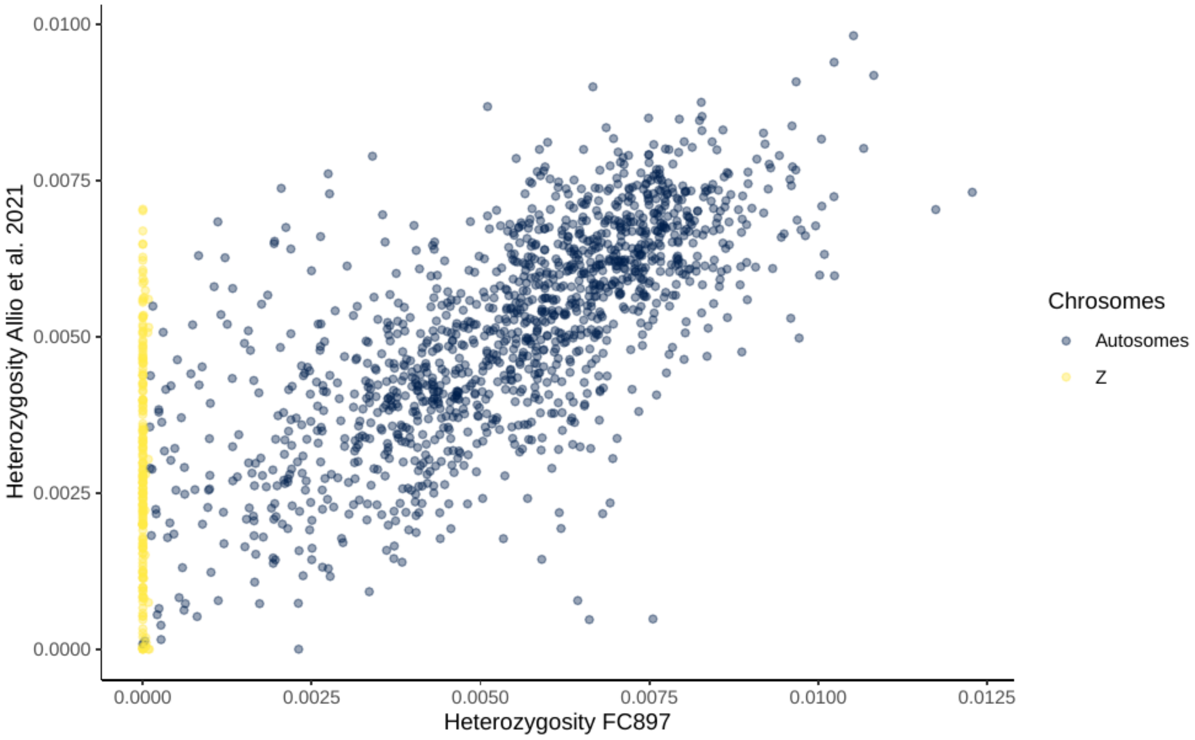

Figure S5. MSMC2 estimates of the effective population size (Ne) with both Illumina and Nanopore data original data or with repeat masked (labeled RM).

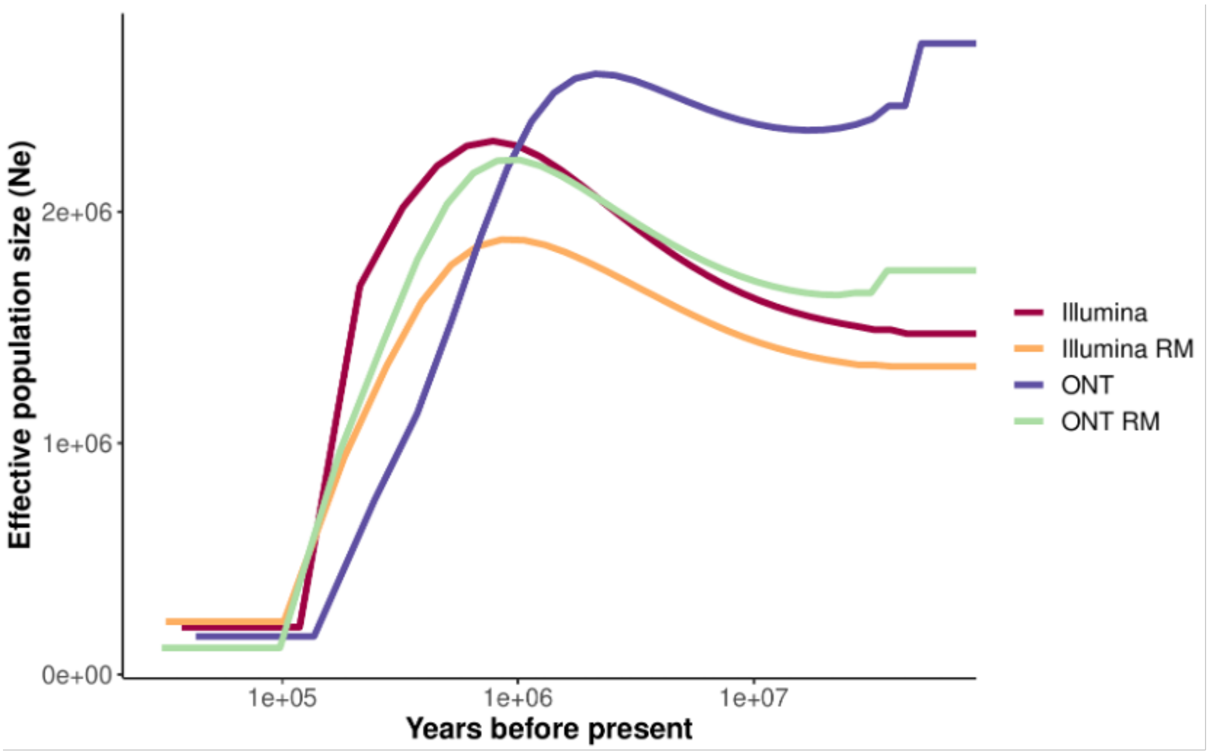

Figure S6. TE copies distribution according to their divergence from consensus across all Papilionini. Past lineage splits are reported for each species and refer to node numbers in Figure 3.

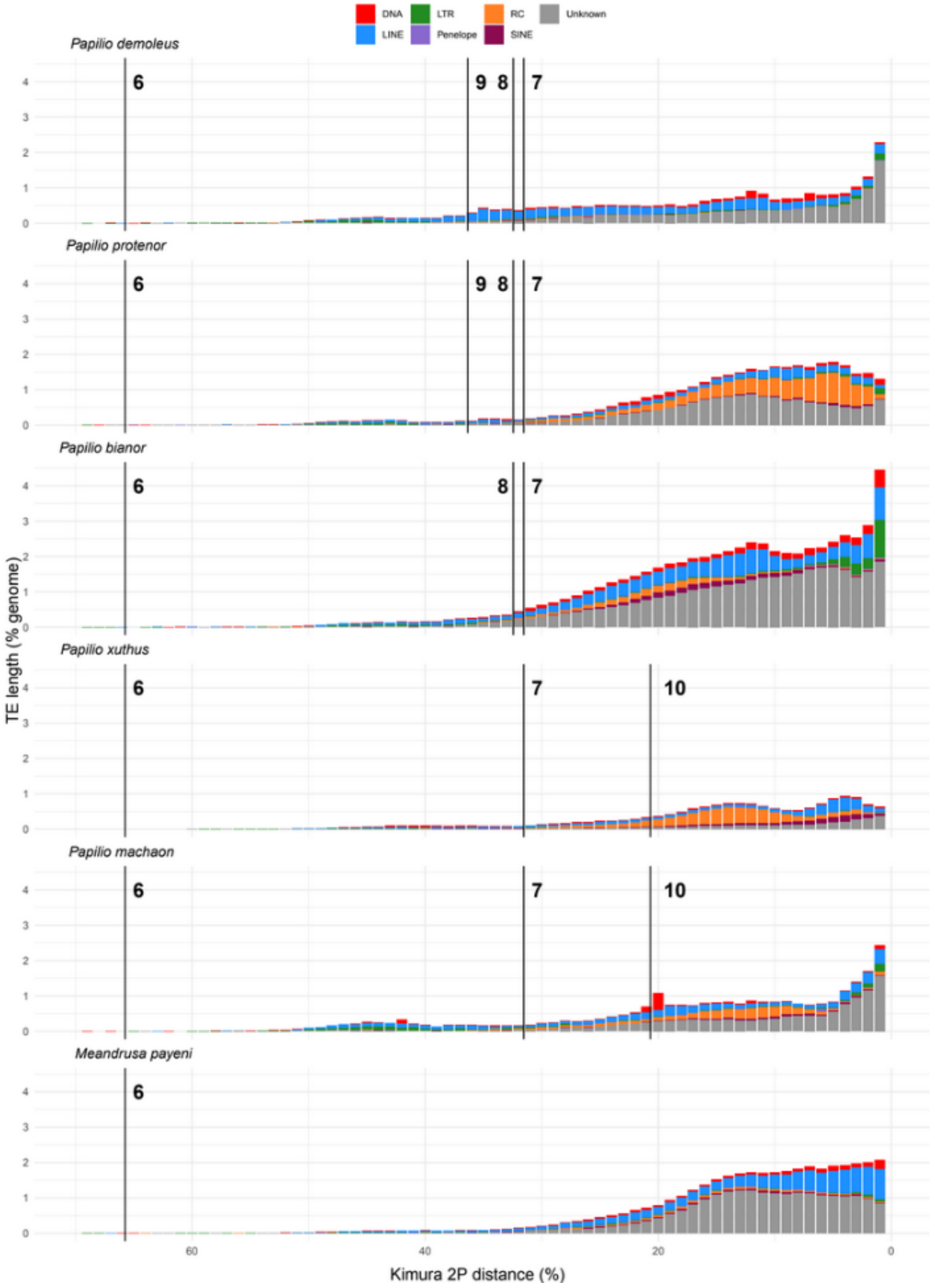

Figure S7. TE copies distribution according to their divergence from consensus across all Troidini and Teinopalpini. Past lineage splits are reported for each species and refer to node numbers in Figure 3.

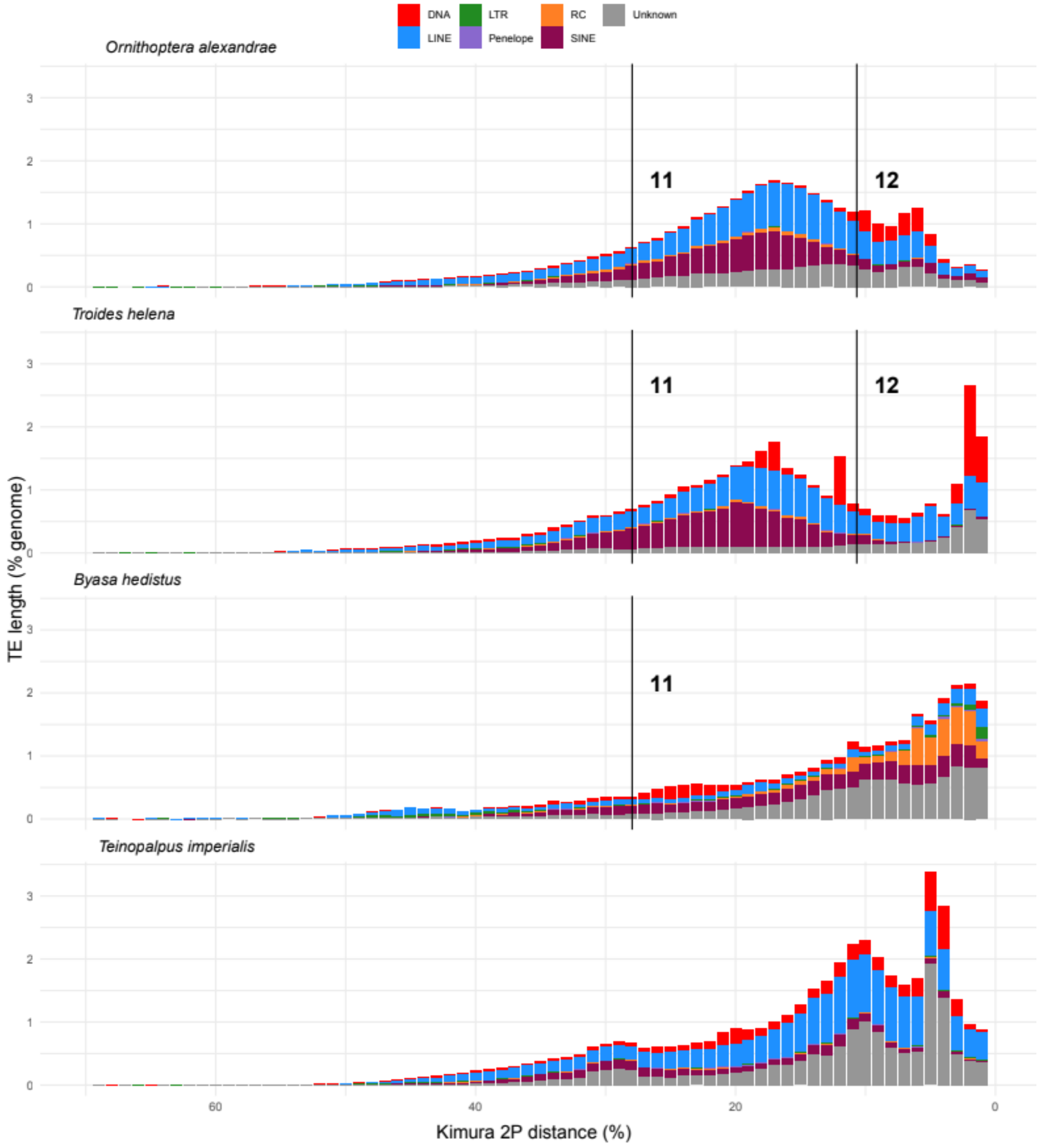

Figure S8. TE copies distribution according to their divergence from consensus across Zerynthiini and Luehdorfiini. Past lineage splits are reported for each species and refer to node numbers in Figure 3.

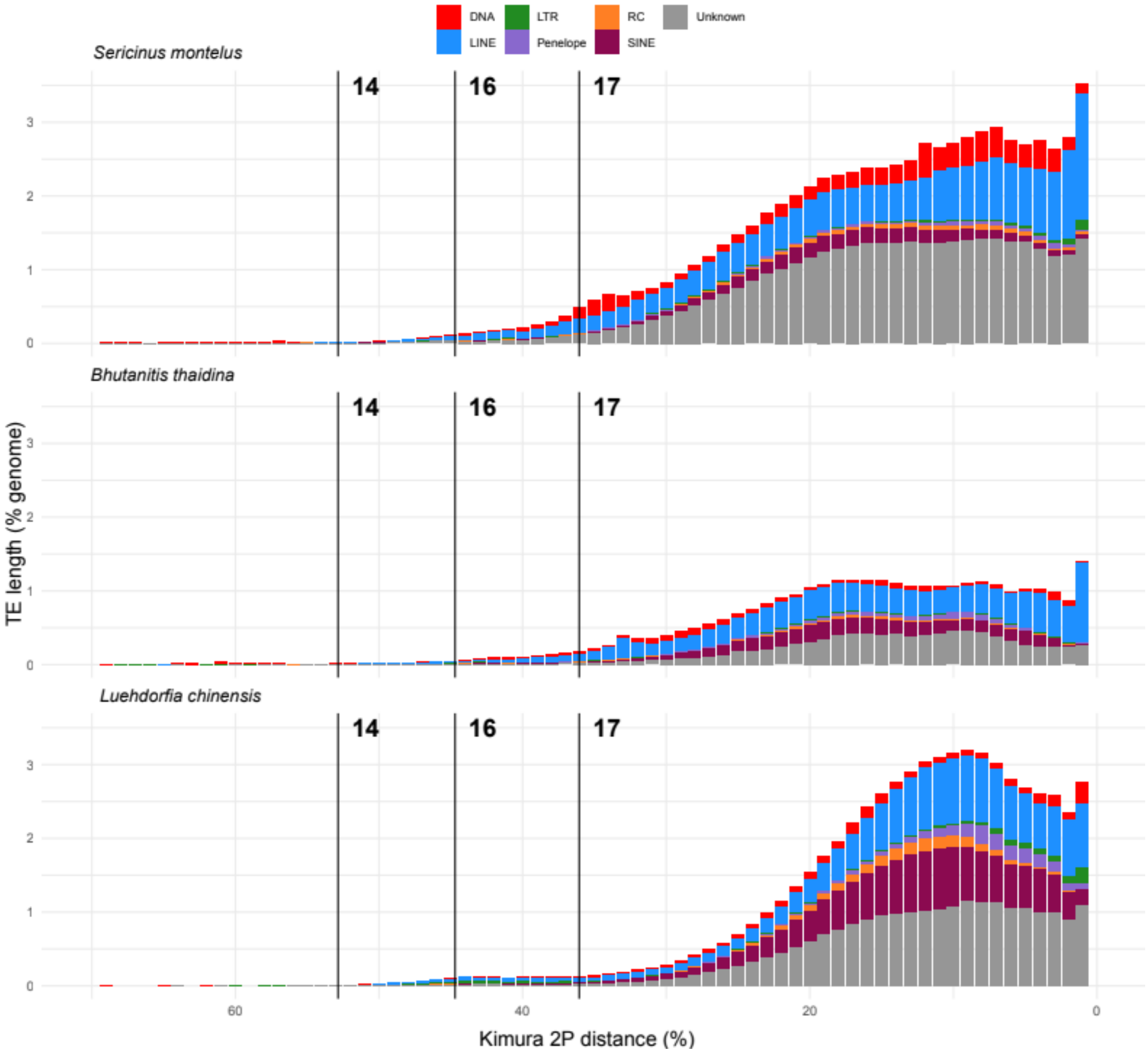

Figure S9. TE copies distribution according to their divergence from consensus across Leptocircini. Past lineage splits are reported for each species and refer to node numbers in Figure 3.

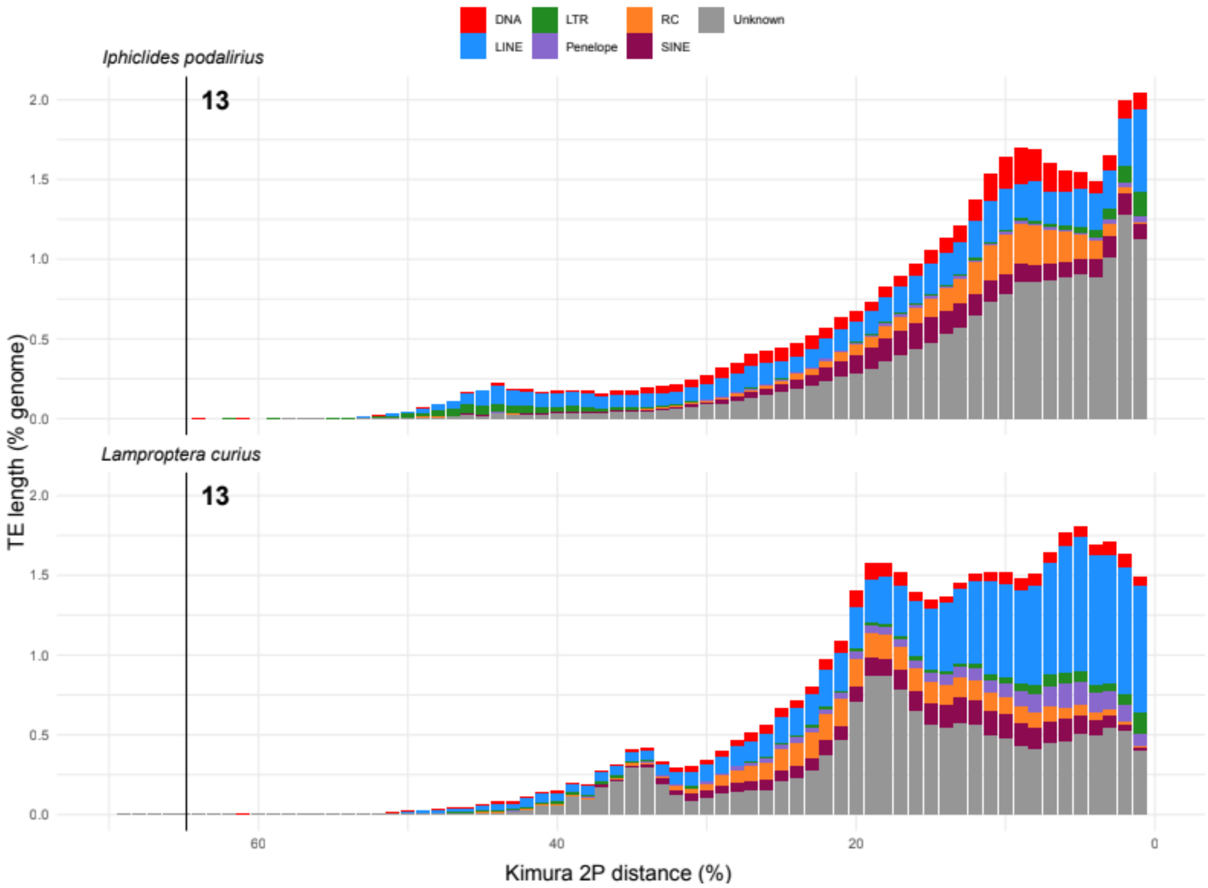

Table S1. Number of contigs removed and their phylum rank (Bacillota, Pseudomonadota) after BlobTools analysis. The total number of contigs removed is 152.

Phylum	Genus	FC897
Bacillota	*Vagococcus*	2
Pseudomonadota	*Citrobacter*	48
	Enterobacter	1
	Enterococcus	8
	Escherichia	9
	Klebsiella	75
	Proteus	1
	Raoultella	1
	Salmonella	7
	**Total Size (bp)**	**15,113,703**

Table S2. Taxonomy, references, assembly sizes and TE annotation results for *Baronia* and the 17 species used in the analysis of GS variation.

Provided as a separate file because of the large size.
